# Role of Intravenous Magnesium in the Management of Moderate to Severe Exacerbation of Asthma: A Literature Review

**DOI:** 10.7759/cureus.28892

**Published:** 2022-09-07

**Authors:** Syed A Bokhari, Shahan Haseeb, Misbah Kaleem, Mohammad W Baig, Haider Ali Babar Khan, Raza Jafar, Shafia Munir, Shawal Haseeb, Zara I Bhutta

**Affiliations:** 1 Internal Medicine, Shifa International Hospital Islamabad, Islamabad, PAK; 2 Otolaryngology, Shifa Tameer-E-Millat University, Shifa College of Medicine, Islamabad, PAK; 3 Shifa Clinical Skills and Informatics Lab (SCIL), Shifa Tameer-E-Millat University, Shifa College of Medicine, Islamabad, PAK; 4 Critical Care Medicine, Shifa International Hospital Islamabad, Islamabad, PAK; 5 Medicine, Rawalpindi Medical University, Rawalpindi, PAK; 6 Internal Medicine, Mayo Hospital, Lahore, PAK

**Keywords:** allergy, literature review, acute asthma managment, intravenous magnesium sulfate, acute asthma

## Abstract

Asthma is a respiratory disorder marked by bronchial irritation and hyperresponsive airway smooth muscle. According to new research, magnesium's dual activity as an anti-inflammatory and bronchodilator may be important in asthma therapy. The goal of this study was to see how effective intravenous magnesium sulfate is in treating severe acute asthma. In addition to checking Clinicaltrials.gov, we ran a database search in Scopus, Google Scholar, PubMed, and Embase. Studies were chosen based on predetermined inclusion and exclusion criteria to prevent the chance of bias. Most researchers believed that intravenous magnesium sulfate improved symptoms and lung function significantly. Mortality and morbidity data were not available.

## Introduction and background

Asthma is a chronic respiratory illness that affects a significant portion of the world’s population [1]. Asthma symptoms include coughing, wheezing, shortness of breath, and an increased respiratory rate. These symptoms are usually mild and may be controlled with medicine and avoidance of known allergens, but they can potentially result in life-threatening exacerbations. Asthma is responsible for an estimated 300,000 fatalities globally each year. The morbidity and mortality from asthma are costly and strain the healthcare system [2].

A potentially life-threatening complication of asthma is status asthmaticus, which results in adverse outcomes for the patient in the short and long term, such as respiratory failure, ICU admission, and sometimes death [3]. Inhaled oxygen, beta-agonists, corticosteroids, and bronchodilators are the mainstays of therapy. Magnesium, a calcium channel blocker, should also be considered since magnesium can aid in the relaxation of constricted bronchioles during an asthma exacerbation. Several studies have associated the onset of asthma with decreased blood levels of magnesium and inadequate nutrient intake [4,5].

Previous research has shown that administering magnesium via intravenous or inhaled routes can help manage acute exacerbations of asthma. Both forms are fast-acting and effective. The intravenous route has greater bioavailability but is associated with systemic side effects [6]. This literature review aims to examine the literature behind magnesium's role in managing asthma.

## Review

**Figure 1:**
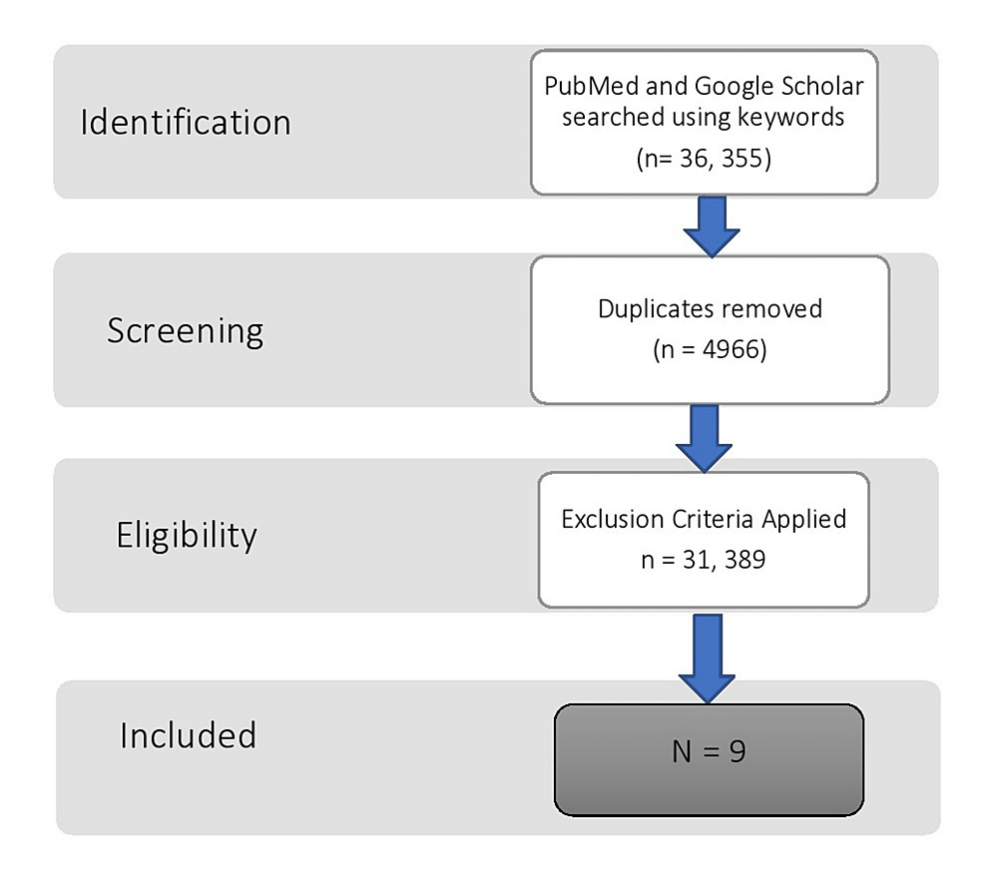
PRISMA framework. PRISMA: preferred reporting items for systematic reviews and meta-analyses.

We followed the preferred reporting items for systematic reviews and meta-analyses (PRISMA) guidelines to screen literature (Figure 1). We reviewed the existing literature using Pubmed and Google Scholar. The keywords utilized in the search were asthma, magnesium, and intravenous. This yielded 255 results on PubMed, which were reduced to 84 when the filter for free full text was applied and only 17 when review articles, systematic reviews, and meta-analyses, were removed. Google Scholar yielded 36,100 hits, of which roughly 7000 were review articles. To further narrow the search, we expanded the keywords to include severe acute asthma, which showed 1240 results, of which 285 were review articles. After applying a custom range from 2012 and narrowing the net to include only clinical trials, 122 studies were shown.

We only included studies that fit the keyword criteria with an adult population published in English. We excluded clinical trials with a crossover design, those in the recruitment process, or those without published results. We also excluded research without full-text options, research on other routes of administration of magnesium such as oral or nebulized, case reports, pre-clinical studies, veterinary studies, non-clinical reports, systematic reviews, and meta-analyses.

A clinical trial by Noppen et al. in 1990 studied the bronchodilating impact of intravenous magnesium sulfate (IVMS) in individuals with severe acute asthma. They concluded that IVMS caused a considerable increase in forced expiratory volume (FEV1) (0.94-0.39 L to 1.03-0.44 L) in ten out of twelve patients and reduced clinical signs and symptoms [7]. An earlier clinical trial by Okayama et al. [8] noted that after IVMS, the bronchodilating response was immediate, and the maximum effect of the IVMS was equivalent to that of an extra albuterol inhalation. Although it resulted in bronchodilation in moderate bronchial asthma patients, the oxygen partial pressure fell in three severe asthma patients. These findings were in contrast to a later clinical trial by Silverman et al. [9], who concluded in their trial that IVMS not only improved FEV1, but the improvement was more significant in patients who presented with poorer FEV1 on admission. However, IVMS did not impact the duration of the in-hospital stay. Another landmark trial to study, the effects of nebulized versus IVMS versus placebo, named the 3Mg trial, concurred with the clinical trial findings conducted by Silverman et al., who concluded that hospitalization rates did not differ between individuals treated with either type of magnesium (nebulized or IV). A scale called the visual analog scale (VAS) was used to quantify the severity of symptoms reported by the patient. The higher the score, the more the severity. The 3Mg study found that while there was no difference in the visual analog scale (VAS) for dyspnea between patients given magnesium and those given placebos, the difference was statistically significant for patients in the IVMS to those in the nebulized group. This meant that while magnesium itself was not superior to placebo, the intravenous form was better than the nebulized form [10]. Tiffany et al. did not find a beneficial effect of magnesium in acute asthma in a randomized, placebo-controlled trial. The participants were segmented into three groups: the first got a 2 g IVMS bolus followed by a continuous magnesium infusion of 2 g/h (16 mEq) over four hours, the second received a 2 g IVMS bolus followed by a placebo infusion, and the third received a placebo bolus followed by a placebo infusion. In this trial, neither the FEV1 nor the peak expiratory volume (PEF) increased after magnesium medication [11].

In 1938, Haury [12] presented important experimental data showing magnesium dilated bronchoconstriction in guinea pigs produced by pilocarpine, histamine, and barium chloride. A second investigation by Haury [13] in 1940 found that half of the patients with severe asthma exacerbations had low blood magnesium levels. IVMS treatment helped two of them improve their clinical conditions, including dyspnea and stridor. The study did not have an ample sample size to extrapolate the findings to the general audience. However, it was one of the first of its kind to test the relationship between magnesium and asthma. In a clinical trial by Bloch et al. [14] in 1995, all patients received IVMS and standard of care, including nebulized albuterol. Additionally, patients with a forced expiratory volume of less than 40% or those with a history of oral corticosteroids within the last six months of the trial were also administered 125 mg of methylprednisolone. After half an hour, patients were randomized to receive either 2 gm of IVMS or 50 ml of saline IV (placebo) in addition to the standard of care for acute asthma. They found that IVMS decreased hospitalization rates and improved FEV in patients with severe acute asthma but had no effect on those with moderate asthma. Green et al. [15] found similar results in their clinical study. Skobeloff et al. [16] conducted a placebo-controlled trial. Participants received either 1.2 g of IVMS in 50 mL of saline for 20 minutes or saline placebo in 50 mL over 20 minutes. Individuals who received IVMS had considerably higher peak flow and lower admission rates than placebo patients (Table 1).

**Table 1:** Study characteristics. FEV1: forced expiratory volume, CHF: congestive heart failure.

Study	Study type	Participants	Inclusion	Exclusion	Primary outcome	Secondary outcome
Noppen et al. [7]	Multiphase clinical trial	Six patients underwent an infusion of MgSO_4_ for 20 minutes	Diagnosed case of bronchial asthma, confirmed via spirometry, aged 45 to 60 years	Patients with heart failure, pneumonia, life-threatening conditions or neoplastic disorders, acidemia or hypercapnia, and patients who require mechanical ventilation	Evolution of FEV1, after MgSO_4_, infusion and after beta-agonist inhalation on two consecutive days	Decrease in wheezing after Mg infusion, subjective improvement in dyspnea, and adverse effects
Okayama et al. [8]	Clinical trial	Two groups: (1) IV saline as the control followed by IV MgSO_4_ (n=5). (2) IV saline as the control followed by an increased dose of IV MgSO_4_ followed by inhaled albuterol (n=5)	Ten randomly selected patients presented with acute asthma	None	Change in FEV1 from baseline and maximum effect compared with that of a beta-agonist	Changes in Mg and albuterol concentrations, improvement in dyspnea, and changes in oxygen partial pressure
Goodacre et al. [10]	Double-blind, placebo-controlled trial	IV Mg (n=396) vs nebulized Mg (n=333) vs placebo (n=358)	Adults (aged ≥16 years) attending an emergency department with severe acute asthma	Patients who had life-threatening features, a contraindication to either nebulized or intravenous MgSO_4_, and individuals who were unable to provide written or verbal consent	Proportion of patients admitted to hospital, either after emergency department treatment or at any time in the subsequent seven days, and patient's visual analog scale (VAS) for breathlessness in the two hours after the start of treatment	Mortality, adverse events, use of ventilation or respiratory support, length of hospital stay, admission to a high-dependency unit, change in peak expiratory flow rate and physiological variables over two hours, change in the quality of life between baseline and one month, and satisfaction with care
Tiffany et al. [11]	Randomized, double-blind, placebo-controlled trial	IV Mg and then maintenance Mg (n=12) vs IV Mg and then placebo (n=15) vs placebo loading dose and placebo infusion (n=21)	Forty-eight asthmatic patients aged 18 to 60 years with an initial peak expiratory flow rate (PEFR) of <200 L/min who failed to double their initial PEFR after two standardized albuterol treatments	Patients with a history of chronic bronchitis or emphysema, oral temperature >38.2°C, history of renal failure, history of CHF, or requiring tracheal intubation should have an initial of PEFR more than 200 L/min	Improvement in FEV1 or PEFR after Mg infusion	None
Haury [12]	Randomized, double-blinded, placebo-controlled trial	IV MgSO_4_ (n=18) vs placebo (n=24)	Forty-two adult patients between the ages of 18-55 with a history of asthma, peak expiratory flow of <100 l/min or 25% of predicted flow, and the ability to give informed consent	Clinical signs and symptoms are consistent with alternate causes of wheezing. Patients who were highly likely to be intubated	Change in peak expiratory flow at 60 minutes	Change in subjective symptoms of dyspnea as measured by the Borg dyspnea scale at 60 minutes and need for hospital admission
Haury [13]	Randomized double-blind placebo-controlled study	Two grams of MgSO_4_ vs placebo in 50 mL of normal saline solution IV. One hundred thirty-five patients total were randomized into two groups	Patients aged 18 to 65 years presenting with acute asthma to the ED	History of congestive heart failure, diabetes mellitus, angina, chronic renal insufficiency, temperature >380°C, pneumonia, or if pregnant	FEV1 at two hours after treatment and hospital admission rates	Follow-up after two hours and once every week for hospital visits for asthma
Bloch et al. [14]	Single-blind, randomized clinical trial	Two-gram IV magnesium sulfate (n=58) vs placebo (n=62)	Patients aged 18 to 65 years with acute asthma are unresponsive to a single albuterol treatment	Patients with, angina, chest pain, uncontrolled hypertension, CHF, metastatic cancer, renal disease, temperature above 38.3°C, systolic blood pressure less than 120 mm Hg, or pregnancy	Change in peak expiratory flow after Mg infusion	Number of patients requiring hospitalization, return to the ED within 72 hours, reactions to Mg infusion
Green et al. [15]	Randomized controlled trials	1.2 gram of IV MgSO_4_ in saline (n=19) vs placebo in saline (n=19)	All patients 18 to 70 years of age presenting to the emergency department at The Medical College of Pennsylvania (Philadelphia) with an acute exacerbation of asthma	Temperature of greater than 38°C, systolic blood pressure less than 120 mm Hg, a history of kidney disease, purulent sputum, infiltrate on a chest roentgenogram, and pregnancy	Hospital admissions and PEFR after intervention	ED treatment duration, ICU admission, hospital length of stay, and vital signs

Discussion

Previous recommendations advocated intravenous magnesium sulfate (IVMS) as a safe and effective therapy choice for adult patients with severe acute asthma who did not respond to first-line medications [17,18]. Magnesium sulfate is a physiological blood coagulation mediator that aids in releasing histamine and acetylcholine, resulting in bronchodilation. Interfering with calcium influx can also cause bronchial smooth muscle relaxation [19]. Another potential mechanism by which magnesium is thought to impact asthma is by dampening the neutrophilic burst associated with asthma leading to an improvement in the signs and symptoms of the disease [20]. According to Mohammed et al. [21], IVMS should be used as a first-line treatment for children with severe acute asthma who have not responded to other treatments. In contrast, the role of nebulized magnesium sulfate in children and the roles of both nebulized and intravenous magnesium sulfate in adults should be further investigated. Due to the low risk of severe side effects from magnesium sulfate, it is permissible to use intravenous magnesium sulfate in patients with life-threatening symptoms when any potential benefit outweighs the risks of treatment [22]. In a study done by Hashimoto et al. in 2000 [23], magnesium concentrations in serum, erythrocytes, and lymphocytes were evaluated in 25 patients with stable bronchial asthma and nine age-matched healthy participants to determine the role of magnesium in bronchial hyper-reactivity. They found that 40% of asthmatic patients had magnesium insufficiency and that the low magnesium concentration in erythrocytes represents lower magnesium storage in bronchial asthma patients. This study supports the usage of magnesium in patients with acute asthma exacerbation. While there is a wealth of information on the hazards and advantages of IVMS, more study is needed to find the best dosage and duration of magnesium administration. Our review had a few limitations: we did not include unpublished material or research that was not published in English, which might have resulted in selection bias.

## Conclusions

The usefulness of magnesium in the treatment of acute asthmatic episodes is unclear. Research and local standards of care prefer to endorse the use of magnesium in severe asthma attacks. Some research supports the use of magnesium in severe asthma exacerbations since IVMS appears to lower inpatient admission rates or hospitalization rates. Also, there are additional research trials where there is an improvement in lung function, which may be the result of IVMS. A randomized, placebo-controlled trial is required to determine the actual benefit of IVMS therapy in patients with severe asthma.
